# Twisting of the Arteries

**Published:** 1831

**Authors:** 


8. *Twisting of the Arteries.*—This is an operation which is intended as a substitute for the usual ligatures, after amputation, &c. M. Amussat, of the Academy of Science in Paris, tried this twisting (torsion) of the arteries after amputation, in four individuals, with entire success; no secondary hemorrhage occurred. It is said never to be followed by consecutive bleedings, and that it allows the immediate reunion of the parts, in due time. The practice has been adopted, with success, by M. M. Waust and Anciause, at Liege; Friecke and Schreuder, at Hamburgh; and Dieffembach and Rust, at Berlin.—*Journ. Royal Inst., May,* 1831.

